# Differentiating cancer cells reveal early large-scale genome regulation by pericentric domains

**DOI:** 10.1016/j.bpj.2021.01.002

**Published:** 2021-01-14

**Authors:** Jekabs Krigerts, Kristine Salmina, Talivaldis Freivalds, Pawel Zayakin, Felikss Rumnieks, Inna Inashkina, Alessandro Giuliani, Michael Hausmann, Jekaterina Erenpreisa

**Affiliations:** 1 Latvian Biomedicine Research and Study Centre, Riga, Latvia; 2 Institute of Cardiology and Regenerative Medicine, University of Latvia, Riga, Latvia; 3 University of Latvia, Riga, Latvia; 4 Environment and Health Department, Italian National Institute of Health, Rome, Italy; 5 Kirchhoff Institute for Physics, Heidelberg University, Heidelberg, Germany

## Abstract

Finding out how cells prepare for fate change during differentiation commitment was our task. To address whether the constitutive pericentromere-associated domains (PADs) may be involved, we used a model system with known transcriptome data, MCF-7 breast cancer cells treated with the ErbB3 ligand heregulin (HRG), which induces differentiation and is used in the therapy of cancer. PAD-repressive heterochromatin (H3K9me3), centromere-associated-protein-specific, and active euchromatin (H3K4me3) antibodies, real-time PCR, acridine orange DNA structural test (AOT), and microscopic image analysis were applied. We found a two-step DNA unfolding after 15–20 and 60 min of HRG treatment, respectively. This behavior was consistent with biphasic activation of the early response genes (*c-fos - fosL1/myc*) and the timing of two transcriptome avalanches reported in the literature. In control, the average number of PADs negatively correlated with their size by scale-free distribution, and centromere clustering in turn correlated with PAD size, both indicating that PADs may create and modulate a suprachromosomal network by fusing and splitting a constant proportion of the constitutive heterochromatin. By 15 min of HRG treatment, the bursting unraveling of PADs from the nucleolus boundary occurred, coinciding with the first step of H3K4me3 chromatin unfolding, confirmed by AOT. The second step after 60 min of HRG treatment was associated with transcription of long noncoding RNA from PADs and peaking of *fosL1/c-myc* response. We hypothesize that the bursting of PAD clusters under a critical silencing threshold pushes the first transcription avalanche, whereas the destruction of the PAD network enables genome rewiring needed for differentiation repatterning, mediated by early response bivalent genes.

## Significance

Differentiation, the process that (starting from identical genetic information across all cells) ends up in distinct patterns of genome expression, is still largely enigmatic. The elucidation of this process holds promises to cure cancer by the induction of redifferentiation of cancer tissues. Breast cancer cells treated with an inducer of differentiation undergo a critical transcription avalanche, starting cell fate change. We hypothesize that this process can be initiated by the rearrangement of the constitutive pericentromere-associated domains (PADs) known by their silencing positional effect. We found that 15 min after differentiation inducer administration, the PADs’ scale-free size distribution bursts into monocentromeric units, losing their silencing activity and synchronizing the genomic DNA unfolding, enable the transcription avalanche leading to genome rewiring starting differentiation.

## Introduction

In human cells and tissues of each individual, the DNA sequence of the genome in each cell nucleus is identical. However, differentiated cells are functioning in their own highly specialized pattern and this means that the genes, although being available in total, must show a different expression level from silenced to highly activated, depending on the tissue functional requirements. This implies a general rewiring of the whole-genome expression network resulting in the specific expression profiles. The most challenging question is, therefore, how the cells start changing their fate in differentiation commitment preceding differentiation itself. The linear genetic information as such cannot provide the answer because it is identical for all cells. Thus, other mechanisms, such as nonlinear thermodynamics of the whole-genome regulation, are required to reorganize the accessibility of genes for transcription (1). We inquired a possible restructuring of the constitutive pericentromere-associated chromatin domains (PADs).

The well-established model breast cancer MCF-7 cell line of the minimally differentiated, little invasive, luminal type A (expressing estrogen and progesterone receptors) (2, 3, 4) was used for the terminal differentiation induction by the treatment with type I neuregulin 1 (also named heregulin (HRG)), a ligand of HER2-positive breast cancer (5). In this model, terminal differentiation is achieved and testified by fat production after a week or more of treatment (6), but here we investigated the very early changes. HRG induces *trans*-activation of ErbB2-receptors through linking to ErbB3, which dimerizes with ErbB2, internalization and clustering of the dimers, and nuclear transport of epidermal growth factor receptor (EGFR) (7, 8, 9, 10). Similarly, neuregulin 1 causes the differentiation of embryonic stem cells into cardiac myocytes (11). In MCF-7 breast cancer cells, 5–10 min after HRG application, the downstream signaling of AKT and ERK kinase pathways, coupled with biphasic induction of the immediate response genes is stimulated (6,12). The earliest FOS gene transcription peaking at 20 min (12) is induced by ERK-mediated activation of its promoter (13). By comparing the ergosterol (ERG) and HRG ligands’ response of MCF-7 cells, in which the first did not induce terminal differentiation and the second did, the respectively transient and sustained activation of ErbB2 receptors (a similar transient and sustained kinetics of activation of AKT and ERK pathways and the similar difference in kinetics of the cascade of the early response genes) were found (6). The needed sustainability of the early response gene network activation lasting for 1–1.5 h is dependent on the sequential induction by FOS of FOSL1 (FRA-1) and then FHL2. Interaction of the last two causes a negative feedback suppression of FOS with gradual downregulation of ERK, whose activation is abated by 3 h.

The authors concluded that both the intensity and duration are ensuring the sustainability of the effective biphasic response of transcription factors and signaling mediators as a condition for initiation of successful differentiation. These were necessary to change secondary gene expression for HRG-induced cellular differentiation of MCF-7 cells, which takes place after HRG- but not ERG-treated MCF-7 cells (6,12,14). Another group (15,16), using the same transcriptome data (22,277 transcripts) after both inducers and evaluating the change of the transcription rate, revealed the early critical acceleration of transcription (avalanche) between 15 and 20 min involving several thousands low-expressed genes and operating with ∼1 Mbp topology-associated-domain (TAD)-sized units. This group also came to a similar conclusion on the necessity of a “genome engine” for starting the HRG-induced cell differentiation, whereas this acceleration of transcription was aborted in the case of ERG stimulation (15,16). These observations were interpreted as proof of the crucial change of the whole-genome superstructure needed for commitment (17). In turn, on the same model, Chen and colleagues (18) revealed a critical, coordinately fluctuating expression of the promoting group of 104 genes at 1–1.5 h. These studies highlighted two points of self-organized criticality (SOC) in the genome transcription in MCF-7-HRG model at 15–20 min and 1–1.5 h after treatment.

The earlier ideas of the self-organizing reconstruction of the genome as a whole (19, 20, 21, 22) were more recently developed by nucleome studies using the chromatin high-structure capture and image analysis. This and a similar approach disclosed cooperative assembly and phase separation of the transcriptionally active and inactive chromatin (23, 24, 25, 26, 27, 28) largely acting on the base of simple physical principles (29). Studies of cell differentiation, often by all-trans retinoic acid from embryonal stem cells, showed an increase of the proportion and nuclear peripheral assembly of the repressed facultative heterochromatin; these studies were set on mature differentiated cells, 4–6 and more days after receiving the corresponding stimuli (28). The effect of pericentric-associated domains (PADs) cosegregating with repressive genomic regions and determining gene silencing in the proximity of centromeres (by position effect variegation) is long known and well established (30, 31, 32, 33, 34). Some reports correspond to the earlier findings on the dynamism of the chromocenters of G1 cells (35) and in postmitotic embryonic stem cells induced to differentiate by all-trans retinoic acid (36). PADs also react by increase of the long noncoding RNA (lncRNA) synthesis to stress conditions (37). However, the dynamic principles of PADs behavior have not been explored at the early stage of differentiation commitment. The earliest term of study was at 24 h (38) when substantial spatial rearrangement and a more dispersed pattern was found for H3K9me3 heterochromatin than in terminally differentiated cells. With a thought that PADs may contribute to critical self-organization of transcription already in the first hour, we set to address it by adjusting the experiments to the time and transcription parameters found in this particular breast cancer MCF7-HRG differentiation commitment model described above. We found scaling behavior of PADs, undergoing bursting simultaneously with the first genome transcription avalanche, starting differentiation commitment.

## Materials and methods

### Cell line and treatments

Cells of the human breast cancer cell line MCF-7 (American Type Culture Collection) were cultured in Dulbecco’s modified Eagle’s media supplemented with 10% fetal bovine serum (FBS; Sigma-Aldrich, St. Louis, MO). Cells were seeded at a density of 2 × 10^4^ cells per well on four well chamber slides and grown in 5% CO_2_ incubator at 37°C. Cell cultures were carried out without antibiotics, tested for mycoplasma, and were taken into experiment only if the apoptotic index was below 5%. For experimental studies, cells were serum starved for 48 h and treated with 50 nM HRG (396-HB (R&D Systems, Minneapolis, MN) or ab50227 (Abcam, Cambridge, UK) for different time points: 10, 15, 20, and 30 min and 1 and 3 h. For ErbB2 receptor clustering and internalization experiments proving the activation of ErbB2 receptors, after 48 h of serum starvation, additionally reduced serum starvation (0.5% FBS) for 3 h was performed. Then the medium was replaced by serum-free medium containing 50 nM HRG for 60 min and finally changed for 10% FBS Dulbecco’s modified Eagle’s media for another 1 h before fixation.

### Acridine orange staining

Cells on chamber slides were washed with warm phosphate-buffered saline (PBS) and pure FBS, and then, after air-drying (5 min) were fixed in ethanol/acetone (1:1) for 1 h at 4°C. After fixation, the chamber slides with control and different HRG treatment points in each slide were passed through decreasing grades of ethanol (96%, 90%, 70%) to PBS. The slides were then treated with RNase A (DNase, protease-free, 10 mg/mL; Fermentas, Vilnius, Lithuania) in PBS for 30 min at 37°C and rinsed in PBS. Then hydrolysis was performed for 40 s at 60°C in 1 M HCl. After hydrolysis, the slides were washed in three changes of distilled water (1 min each), passed through PBS for 2 min and 0.1 M acetic buffer (pH 4.1) for 5 min. Staining with the high-purity acridine orange (AO) (Polyscience, Niles, IL) at a concentration of 10^−4^ M was performed in the same buffer for 15 min (in the dark) following three changes of AO diluted to a concentration of 10^−6^ M in the same acetic buffer, sealed in the latter solution under a glass coverslip with nail polish and examined within 2 h.

The images of cell nuclei were acquired in each chamber slide of each experiment using an epifluorescence microscope Leica DM LS2 equipped with both a Sony DXC-S500 color video camera and a Leica 40×/NA 0.65 objective. The fluorescence filter cube Leica I3 was used (excitation filter BP, 420–490 nm; dichromatic mirror, 510 nm; suppression filter LP, 515 nm). Image analysis and quantification of the red/green index (R/G) of AO staining was performed using the ImagePro Plus 4.5 software (Media Cybernetics, Rockville, MD). The color images recorded by the video camera via the I3 optical filter cube was digitally separated into a red and green color plane and converted into grayscale pixel values, and the integral optical density was measured for each plane. Integral optical density corresponds to the integrated fluorescence intensity (IFI) and the R/G was thus calculated as the ratio of the two IFI (= IFI_red_/IFI_green_).

A total of 260–450 cells were microscopically imaged at each time point, including serum-starving (ST) control on each chamber slide tested for 20 min and 60 min of HRG treatment. The cells for three AO test experiments were subcultured from passages 7 and 8.

### Immunofluorescence

Cells on chamber slides were washed with warm PBS and fixed in 4% paraformaldehyde/PBS for 15 min at room temperature. Slides were then washed thrice in PBS 0.1 M glycine for 5 min, once in PBS 0.2% Triton X-100 for 5 min, and once in PBS for 5 min. Slides were subsequently blocked for 15 min in TRIS-buffered saline (TBS), 0.025% Tween 20, and 1% bovine serum albumin at room temperature. Specimens were covered with a blocking solution containing the primary antibody (Table 1) and incubated in a humidified chamber at 4°C overnight. Specimens were then washed thrice in TBS, 0.01% Tween 20 and covered with appropriate secondary antibodies (goat anti-mouse IgG Alexa Fluor 488, goat anti-rabbit IgG Alexa Fluor 594; Invitrogen, Carlsbad, CA) and incubated for 40 min at room temperature in the dark. Slides were washed thrice for 5 min with TBS and then counterstained with 0.25 *μ*g/mL DAPI for 2 min, and finally embedded in Prolong Gold (Invitrogen). The characteristics of used antibodies are presented in Table 1.

**Table 1:** The antibodies used, their specificity, and source

Antigen	Description	Specificity/immunogen	Dilution	Product-Nr and manufacturer
c-ErbB-2	Mouse monoclonal	The purified extracellular domain of human ErbB2 from transfected cells	1:100	E2777; Sigma-Aldrich, Munich, Germany
Centromere Protein CENPA	Human	Derived from human CREST patient serum	1:50	15–234; Antibodies, Davis, CA
H3K9me3	Rabbit polyclonal	Synthetic peptide within human histone H3 aa 1–100 (N-terminal) (tri methyl K9)	1:200	ab8898; Abcam, Cambridge, UK
H3K4me3	Mouse monoclonal	A synthetic peptide corresponding to human histone H3 aa 1–100 (trimethyl K4)	1:100	ab1012; Abcam, Cambridge, UK

### Microscopy and image analysis (except AO analysis described above)

For microscopic observations after immunofluorescence (IF) staining, a fluorescence light microscope (Leica DM LS2; Leica, Wetzlar, Germany) equipped with a color video camera (Sony DXC S500; Sony, Tokyo, Japan) and laser scanning confocal microscopes (LEICA TCS SP8 and LSM710 (Zeiss, Oberkochen, Germany)) were used. To capture epifluorescent images, a 100×/NA 1.25 objective in addition to separate optical filters and a three-band blue-red-green (blue, red, green excitation filter: BP 400/30, BP 495/15, BP 570/20; suppression filter: BP 465/20, BP 530/30, BP 640/40) optical filter (Leica) for IF slides were used.

#### Image quantification

*DNA content measurement of DAPI-stained chromatin.* Under the same exposure conditions, cell nuclei were imaged by the color video camera of the epifluorescent microscope using appropriate DAPI filter settings. The obtained images were converted into monochromatic gray-level images and the contours of the nuclei were determined by visual inspection. The numbers of pixels and the integrated DAPI fluorescence intensity inside the closed contours were determined.

*Area/count of chromocenters in PADs.* Areas of H3K9me3-positive chromocenters (red-stained) were determined as the number of pixels counted in the red color plane inside the visually selected contour of the DAPI-stained nucleus (blue color plane). The areas and numbers of centromeres per nucleus were registered.

*Heterogeneity analysis of the euchromatin distribution in cell nuclei.* The heterogeneity of the H3K4me3-stained transcriptionally active chromatin was determined inside the visually selected DAPI contours of the cell nuclei as fractions of pixels that deviate more than 10% from the average fluorescence intensity in the green image channel.

*Cluster analysis of centromeres in PADs.* Clustering was calculated using a function in ImagePro Plus. The number of centromeres was counted by the number of green immune-stained centromere-associated-protein (CENPA)-positive areas inside selected red H3K9me3-positive chromocenters whose contours were also determined interactively. The automatic centromere counts were controlled by visual inspection and correction.

#### Superresolution imaging

For further illustration, superresolution single-molecule localization microscopy was used as described in detail in (10,39). The instrument was equipped with a 100×/NA 1.46 oil immersion objective lens. A solid-state laser (491 nm) was used for inducing molecular blinking and 2000 image frames were detected by an iXon Ultra Andor EMCCD grayscale camera. For visualization, next neighbor images were prepared, i.e., the intensity of each point correlating to the number of neighboring points in given surroundings.

### Real-time PCR

The total RNA was extracted from 10^6^ MCF-7 cells by using TRIZOL (Invitrogen). First-strand cDNA was synthesized using 2 *μ*g of RNA, random hexamers, and RevertAidTM M-MuLV Reverse Transcriptase (Fermentas) according to the manufacturer’s protocols, and subsequently diluted with nuclease-free water 10 times. Real-time PCR (qPCR) was run on a ViiA7 (Applied Biosystems, Waltham, MA). Amplification mixtures (25 *μ*L) for all amplicons contained 2 *μ*L template cDNA, 2X SYBR Green Master Mix buffer (12.5 *μ*L) (Thermo Fisher Scientific, Vilnius, Lithuania) and 100 nM forward and reverse primer. The primer sequences are presented in Table 2.

**Table 2:** The primers applied in qPCR

Gene	Forward primer sequence	Reverse primer sequence	Reference
FOS	ACTTGAAAGCATCCATGTGTGTGGAC	GGCCTGGCTCAACATGCTACTAA	(12)
FOSL1	AGCAGCAGCAGGTGATTGGA	CGCAGATCAGCTCATCACAGAAG	(12)
FHL2	TGGCATAACGACTGCTTTAACTGT	GTGTGAGATCACAAGCAGCAA	(12)
MYC	TGCTCCATGAGGAGACACC	CGTAGTTGTGCTGATGTG	in house
GAPDH	GGGTCTTACTCCTTGGAGGC	GTCATCCCTGAGCTAGACGG	(40)
B2M	TCTCGCTCCGTGGCCTTAGC	GCCTACCTACTTTGGGTCTGTGT	(40)
GAPDH v.2	CACCATCTTCCAGGAGCGAG	CCTGCAAATGAGCCCCAGCC	(41)
HS3-9	AATCAACCCGAGTGCAATCGAATGGAATCG	TCCATTCCATTCCTGTACTCGG	(41)
HS3-1	AGTCCATTCAATGATTCCATTCCAGT	AATCATCATCCAACGGAAGCTAATG	(42)

The cycling conditions for the FOS, FOSL1, FHL2, MYC, GAPDH, and B2M comprised 10 min of polymerase activation at 95°C and 40 cycles at 95°C for 15 s and 60°C for 60 s. A melt curve was also performed after the assay to check for the specificity of the reaction. This consists of 20 s at 72°C followed by a ramp-up of 1°C steps with a 5-s hold at each step. Each cDNA sample was normalized against two housekeeping genes, GAPDH and B2M, by geometric averaging using geNorm software (40). The calculated gene expression stability coefficient M was applied to qPCR results.

The cycling conditions for the GAPDH v.2, HS3-9, and HS3-1 comprised 10 min of polymerase activation at 95°C and 40 cycles at 95°C for 15 s, 57°C for 20 s, and 60°C for 50 s. The need for a second pair of GAPDH primers arises because of different optimal annealing temperatures for HS3-9 and HS3-1 primer pairs. A melt curve was also performed after the assay to check for the specificity of the reaction using the same conditions as above. Expression was normalized to the GAPDH using the formula:

ΔCT=CT(HS3−9orHS3−1)−CT(GAPDH).

RQ and *ΔΔ*CT were calculated inside each qPCR plate by the following formula:

$$\Delta \Delta CT=\Delta CT-\Delta CT\left(ST\;control\right),\;RQ=2^{-\Delta \Delta CT.}.$$

### Statistical analysis

Comparison between groups was performed by two-tailed *t*-test analysis. Obtained *p*-values were adjusted for multiple test correction applying the Benjamini and Hochberg method (43). We preferred the use of different independent *t*-tests to the analysis of variance strategy in consideration of the context dependence of analysis of variance that, in all cases in which only one of the studied groups is expected to be significantly departing from controls, can severely bias the obtained results. Actually, post hoc analyses imply the presence of a global statistical significance of among groups variance (44).

## Results

We set to address PAD’s topology changes in the MCF-7 cells grown, stimulated, and fixed in situ on the chamber slides for the analysis at the early terms after treatment with HRG, around just the same time points, when critical phase transitions of the genome transcription have been revealed. To be sure in the reproduction of the model, we first checked the sustainability of the binding and clustering of ErbB2 receptors and the timing of biphasic gene activation at the early time points of HRG treatment.

### Activation of ErbB2 receptors and biphasic early genes response by HRG

**Figure 1:**
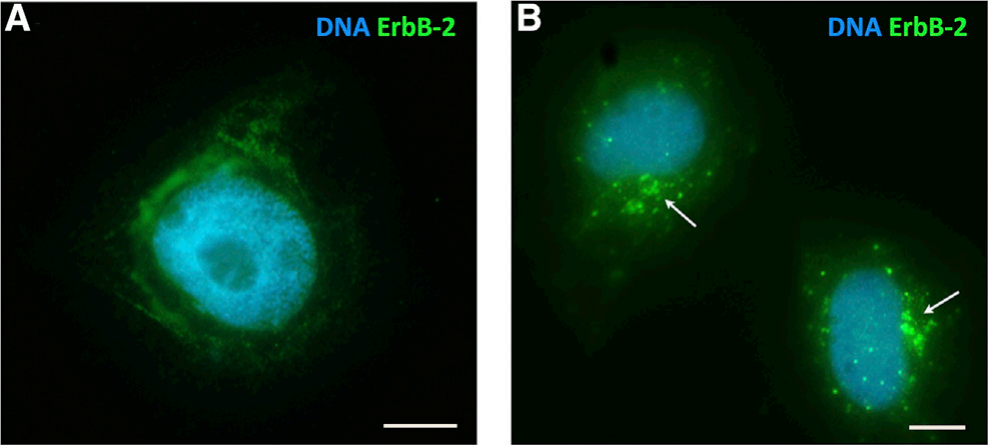
Verification of the MCF-7-HRG model by IF staining of ErbB2 receptors: (*A*) ST control; (*B*) cytoplasmic internalization and clustering of the ErbB2-positive foci after treatment with 50 nM HRG for 60 min. These data are indicative of two independent experiments and four technical replicates. Scale bars, 10 *μ*m.

For activation and visualization of ErbB2 or ErbB2/ErbB3 dimerization, respectively, the procedures and the antibody were used as described in (10). After treatment with 50 nM HRG tested at 60 min, we obtained clustering of ErbB2 receptors, their transition from the cell surface, and aggregation in the cytoplasm in the cells (Fig. 1, *A* and *B*).

**Figure 2:**
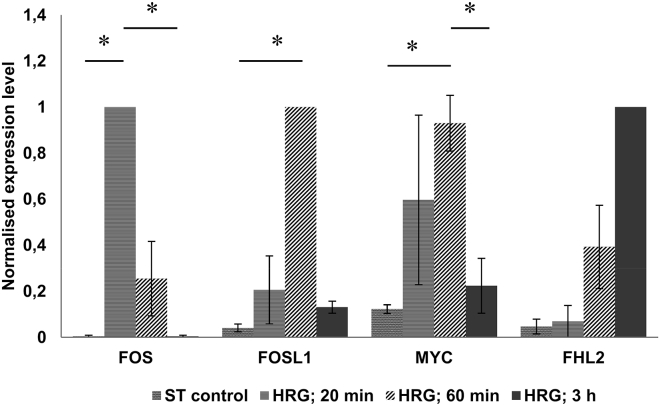
Biphasic expression of the early response genes evaluated by qPCR at four time points normalized to the maximal averaged expression (1 unit on *y* axis) of each of the four tested genes (to better visualize the dynamics of response). The average values of two (three for MYC) independent experiments with standard deviation and significant *p*-values (except FHL2) adjusted by multiple *t*-test corrections are shown.

By qPCR, we next checked the activation and dynamics of the transcription of the key genes of the early gene response at 20 min, 60 min, and 3 h. As presented in Fig. 2, we were able to reproduce the biphasic kinetics reported by the Hatakeyama group (12). Actually, we demonstrated a very high upregulation of FOS by 20 min, with the following peak of FOSL1 at 60 min ending with the downregulation of both genes at 3 h. The FOS dynamics went together with the opposite time course of the FHL2 gene (the third gene of this group), thus revealing a negative feedback loop. MYC, which is known to be activated by FOS (12,45), was reaching an activation peak together with FOSL at 60 min after HRG treatment (Fig. 2).

Therefore, we validated the MCF-7-HRG model acting in our hands with the same effect and time-schedule of the previous studies. Moreover, we demonstrated, consistently with Tsuchiya et al. (15,16), the presence of critical phase transitions (two avalanches) of the whole transcribed genome at 15–20 min as well as the 60–75 min transition reported by Chen et al. (18).

Thus, we decided to follow the DNA conformation at both terms using a cytophysical approach (the DNA AO in situ test) to check for its temporal consistency with gene expression results.

### HRG-induced two-step-DNA-unfolding revealed by the DNA structural AO test at 20 and 60 min

AO is a metachromatic fluorophore that binds to nucleic acids in two forms: intercalating monomers (fluorescence absorption maximum at 502 nm and emission maximum at 530 nm) and coparallel dimers electrostatically attached to the phosphate residues of the DNA backbone (fluorescence absorption maximum at 460 nm and emission maximum at 640 nm). After RNA extraction, the conformation changes correlate to the DNA double- and single-strand transitions (46) or rather, the interplay between a secondary DNA structure and tertiary structure with looser- or tighter-bound histones. This difference in packaging is provoked by short acid pretreatment and can be detected by changes of fluorescence spectra of the dye. This test is very sensitive and applicable for various purposes (47, 48, 49, 50, 51, 52). The R/G (fluorescence intensity ratio of 640/530 nm) is used as a physical parameter of the DNA conformation (51).

**Figure 3:**
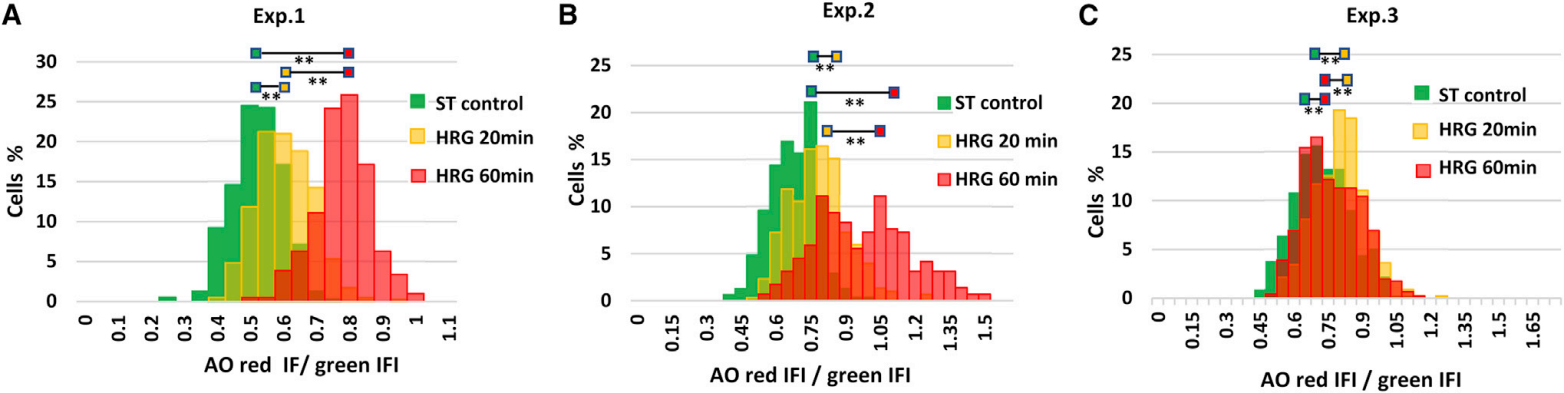
DNA unfolding induced after 20 and 60 min of HRG treatment related to nontreated ST control as revealed by the AO DNA structural test in three independent experiments. Although the 20 min R/G shift occurred in all experiments, the 60-min peak significantly differed in their positioning; thereby, (*B*) was separated in the second experiment for less and more promoted subpopulation. In the third experiment, with the smallest 20-min shift, the 60-min chromatin state was apparently unstable and most cells returned to nearly control position. The *p*-values were adjusted by multiple *t*-tests correction approaches.

The histograms of DNA AO test from three independent experiments characterizing the behavior of the whole cell population are shown in Fig. 3, and the relative synthetic statistical descriptors are presented in Table 3.

**Table 3:** Changes of R/G in DNA AO structural test in the dynamics of HRG treatment found in three independent experiments

Exp. No. (number of cells for each time point)	R/G ST control average ± STD	R/G HRG 20-min average ± STD (*p*-value)	R/G HRG 60-min average ± STD (*p*-value)	*p*-value R/G HRG 20 min versus R/G HRG 60 min
Exp 1 (400)	1 ± 0.15	1.16 ± 0.16 *p* < 0.001	1.49 ± 0.16 *p* < 0.001	*p* < 0.001
Exp 2 (260)	1 ± 0.15	1.16 ± 0.18 *p* < 0.001	1.44 ± 0.30^a^*p* < 0.001	*p* < 0.001
Exp 3 (450)	1 ± 0.17	1.11 ± 0.16 *p* < 0.001	1.06 ± 0.17 *p* < 0.001	negative *p* < 0.001

Average values of R/G were compared and normalized to the average values of the starving control (ST) in each chamber slide (one chamber slide for each row); *p*-value adjusted by multiple *t*-test correction.

a The presented average is composed of the two discrete peaks: the second peak is more significantly shifted ahead (R/G = 1.62), please, see in Fig. 3*B*.

We found a statistically significant R/G shift for more open DNA conformation 20 min after HRG treatment in all three experiments and a further shift of the DNA unfolding at 60 min in two out of three experiments (Fig. 3; Table 3). The R/G shift averages for 60 min in the first and second experiment were similarly reaching near 1.5-fold of the control (Fig. 3, A and B; Table 3); however, in the second experiment, the 60-min fraction clearly separated in two peaks (Fig. 3 B): one at the distal fringe from the 20-min position and another, more promoted, relative to a sort of Avangard cell subpopulation (R/G = 1.62). In the third experiment, in which the 20-min shift was significant but the smallest, the cells have not shifted further at 60 min after HRG but returned the DNA conformation closer to the control (Fig. 3 C; Table 3). So in this particular experiment, the conformation change achieved by a too-small first shift turned out to be not sustainable.

For the summary, in the AO test studies, we found, in three independent experiments, two shifts of the DNA unfolding after HRG action: the first, mandatory at 20 min and the next, after 60 min for more open chromatin conformation, which very likely depended on the cooperative energetic state of the whole cell population achieved in the first shift.

### H3K9me3-chromocenters containing centromeres (PAD) display highly heterogeneous distribution in ST controls

**Figure 4:**
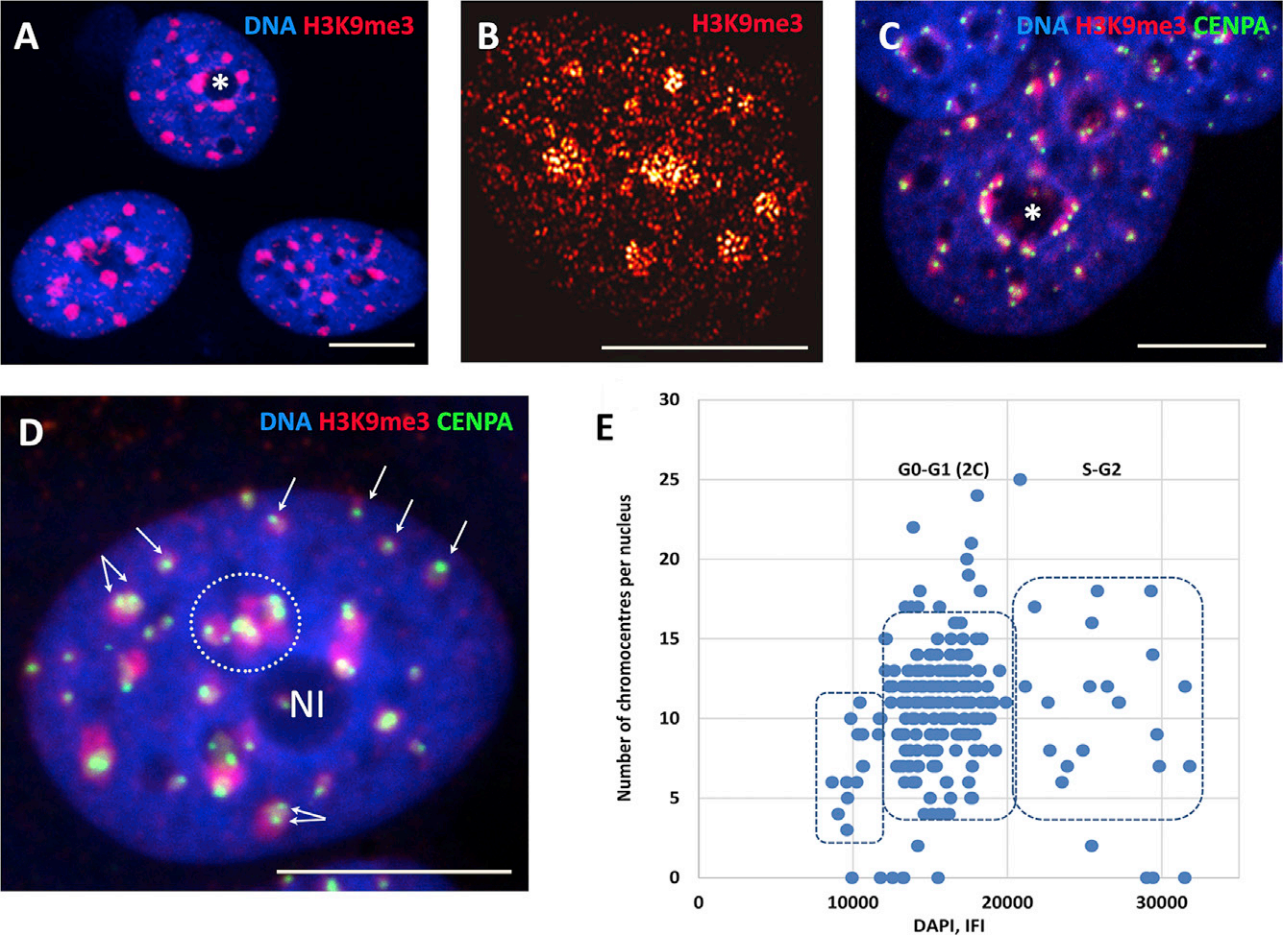
(*A*) Epifluorescent microscopy image of a cell nucleus with H3K9me3-positive chromocenters being strongly clustered around nucleoli (*asterisk*), the nucleus DNA is counterstained with DAPI; (*B*) H3K9me3-positive chromocenters are represented by high-density regions (*bright in the next neighbor image presentation*) in single-molecule localization microscopy; (*C*) confocal section of cell nuclei costained for H3K9me3 and CENPA, identifying the PADs, and the nucleus DNA is counterstained with DAPI; (*D*) confocal maximum projection image costained for H3K9me3 and CENPA with large PAD clusters (*encircled*) near nucleolus (*Nl*) and small PADs (*arrowed*). Scale bars in panels (*A*)–(*D*), 10 *μ*m. (*E*) H3K9me3 chromocenter numbers versus their size in ST control before adding of HRG; the *y* axis signifies the number of H3K9me3-positive chromocenters in individual cells, and the *x* axis signifies the DNA content in these cells as measured by DAPI IFI; the boxed information are as follows: the box to the left shows preapoptotic cells, the box in the center shows the main cluster of 2C cells with highly heterogeneous chromocenter numbers, and the box on the right shows the scarce S-G2 cells.

For IF imaging, the MCF-7 cells were stained with an antibody against the histone H3 three-methylation site H3K9me3, a PAD-specific repressive mark (34). Typical epifluorescent microscopy results of ST control cells are presented in Fig. 4 *A*. For comparison, the pointillist pattern of the H3K9me3 distribution obtained by single-molecule localization microscopy is presented (Fig. 4 *B*), indicating high-density regions compatible with epifluorescence results. In Fig. 4 *C*, a characteristic pattern of a confocal section is presented in which H3K9me3 and the centromere protein CENPA were costained; in the maximal projection image of a confocal stack, a similar pattern is seen (Fig. 4 *D*). The signals are clearly colocalized. As can be noted in Fig. 4, *C* and *D*, the PADs show two distinct topologies: on one hand, a tendency to clump, as a few located near-around the nucleoli, and on the other hand, a tendency to disseminate, as many small clusters up to monocentromeric PADs toward the nuclear periphery.

Epifluorescent images were used for the evaluation of the averaged number and size of the chromocenters per cell. For the evaluation of PAD’s size and numbers, we had to know the cell cycle of the (ST) control. In a few experiments, the specimens stained for PAD counts with antibodies against H3K9me3 and counterstained with DAPI for DNA content evaluation were analyzed on microscopic slides by in situ image analysis and cytometry. The representative distribution for individual cells is shown in Fig. 4 *E*. Most cells contain the 2C DNA content (determined in ana-telophases of the nontreated control, data not shown), which means that they are in the G0-G1 phase (*middle box*). About 5% of the cells show a preapoptotic aggregation of the chromocenters with some DNA loss (*left box*), whereas ∼9–10% of the cells are in S-G2 phase (*right box*) because this cell line, although being mostly delayed in the G0-G1 phase by serum starvation, never fully stopped the cell cycle. In nearly all experiments performed with this model for different purposes (more than a dozen), we used control of DNA in situ staining by Toluidine blue for microscopic inspection. It always revealed, without exception, the presence of low-level mitotic activity after even 48 h of serum starvation, which we chose to apply before HRG treatment. Still, as seen in Fig. 4 *E*, the majority of ST control (∼85%) resides in the 2C fraction and thus, their chromocenter (PAD) numbers and area are both not a subject of DNA replication. Despite this, following the distribution along the *y* axis of the major 2C fraction (*middle box*), one can see that these cells contain a very heterogeneous average number of H3K9me3-positive chromocenters per nucleus; the majority varying between 6 and 16.

### PAD size, numbers, and centromere clustering in the ST control: a scale-free relationship between number and size of PADs

**Figure 5:**
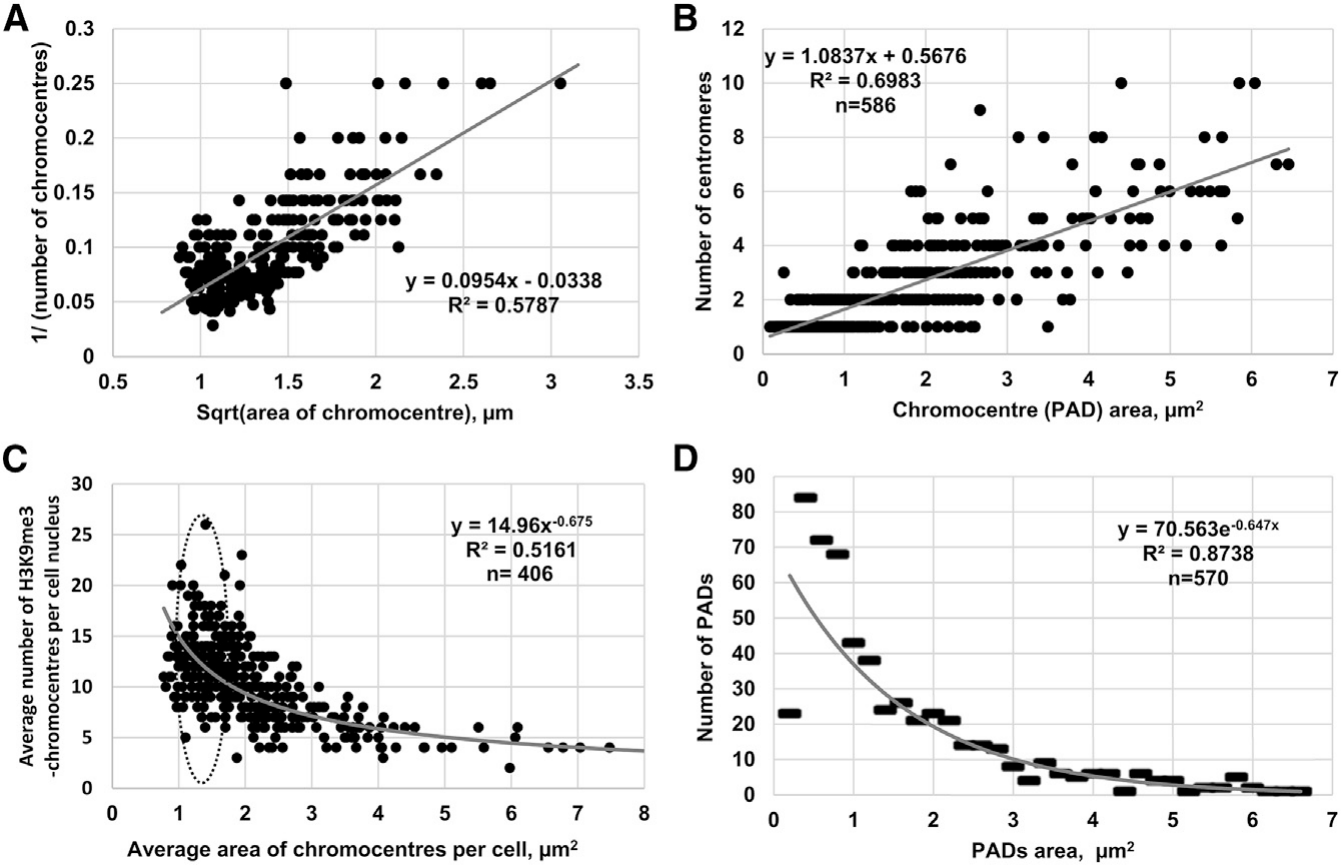
PAD numbers, sizes, and centromere clustering in ST control cells. (*A*) The relationship between the reciprocal value (n^−1^) of the average unraveled a number of the H3K9me3-chromocenters per cell (reciprocal value on the *y* axis) and a proxy of their perimeter (square root of chromocenter area on the *x* axis) from merged data of three independent experiments. The 1/n power-law scaling with the size of the chromocenters is a signature of scale-free distribution (lack of a characteristic chromocenter size), indicating that the chromocenters change their numbers in a cell by splitting and fusion the constant proportion of the constitutive pericentromeric heterochromatin. (*B*) the number of centromeres versus individual PAD area; the distribution follows a linear relationship indicating centromere clustering in PADs of ST control cells. (*C*) The power-law distribution of PAD’s average number versus average size per cell recorded by epifluorescence microscopy resolution in 406 ST control cells, an area of critical transition is encircled; (*D*) the number of individual PADs versus their area follows an exponentially decreasing relation, mostly embracing the PADs with the size below 5–6 *µ*m^2^.

In three independent experiments, we further deliberately selected cells from the ST controls: those that possessed mostly small PADs and those with mostly large ones (excluding the frankly preapoptotic cells). This mixed set (406 cells) was evaluated for the average number of both parameters, PAD number and PAD size per cell nucleus, which are presented in Fig. 5 *A*. In turn, maximal projection confocal images (see for example in Fig. 4 *D*) were used for the study of individual PADs in individual cells; the dependence of their clustering on centromere numbers (with the application of CENPA costaining) is presented in Fig. 5 *B*.

In Fig. 5 *A*, the relationship between the inverted number of PADs (*y* axis) and their linear size (represented by the square root of their area, *x* axis) averaged per cell is shown. The high fit (R^2^ = 0.58) of the values in the positive linear model demonstrates that small and large chromocenters could arise only by splitting and fusing of the same material, corresponding to the constitutive, constant proportion of the pericentric heterochromatin in cell nuclei. In Fig. 5 *B*, the results of the cluster analysis of the centromeres (labeled by CENPA antibody) related to the area of 586 individual PADs (labeled by H3K9me3 antibody) of the ST control are presented for 22 randomly selected cell nuclei.

One can see that although the individual PADs containing one centromere largely differ in their area (because of the difference of the chromosome-specific heterochromatic portion of the PAD size (33)), on the whole, the centromere number in PADs is positively correlated with the heterochromatin area where they occupy. Both representations describing a different measure of PAD numbers versus PAD areas reveal similar correlations (Fig. 5, *A* and *B*). For Fig. 5 *B*, this distribution by the linear model of correlation (R^2^ = 0.70) equally well fitted the positive exponential (R^2^ = 0.69) sloping toward large clusters with more than six centromeres (the formula and trendline not shown). It suggests some deterministic boundary of a size limit for large PAD clusters that, as being presented in Fig. 4, *C* and *D*, is likely topologically created by the nucleolar organizers (NORs). Nevertheless, both distributions of the averaged PADs from preselected 406 cells gathered in three experiments and clustering of centromeres determined in 586 individual PADs of only 22 randomly gathered cells of one experiment are surprisingly similar. We conclude that the PADs are redistributing the same portion of constitutive pericentric heterochromatin by clustering and unclustering (fusing and splitting). This is evident from Fig. 5 *C*, in which the relationship between the PAD number and size (expressed as both single-chromocenter area and cell area occupied by chromocenters) follows a power-law distribution.

Both power-law and exponential distributions are consistent with a scale-free behavior that in synthesis implies the absence of a PAD “characteristic size” and consequently the absence of a bell-shaped symmetric distribution of PAD sizes around its mean value (53).

Although in Fig. 5 *C* the PADs are averaged (unified and thus reveal themselves only as functional units), the distribution in Fig. 5 *D* is different. The PADs are individualized and resolved by confocal microscopy in smaller units (up to 0.2 *μ*m^2^). After removing all the zero values on the *x* axis, this distribution (Fig. 5 *D*) perfectly fits an exponential law (R^2^ = 0.87). Both distributions (Fig. 5, *C* and *D*) are negative and suggest a scale-free sizing, in agreement with a splitting-fusion dynamics.

### Unraveling of PADs after HRG stimulation coincides in time with the first step of DNA unfolding

Our next question was, how this heterogeneity of PADs in ST control cells was changed after HRG treatment, where the first transcription acceleration avalanche was revealed between 15 and 20 min HRG treatment (15,16) and when we also have recorded the mandatory nuclear DNA conformational shift by the AO test in three independent experiments.

**Figure 6:**
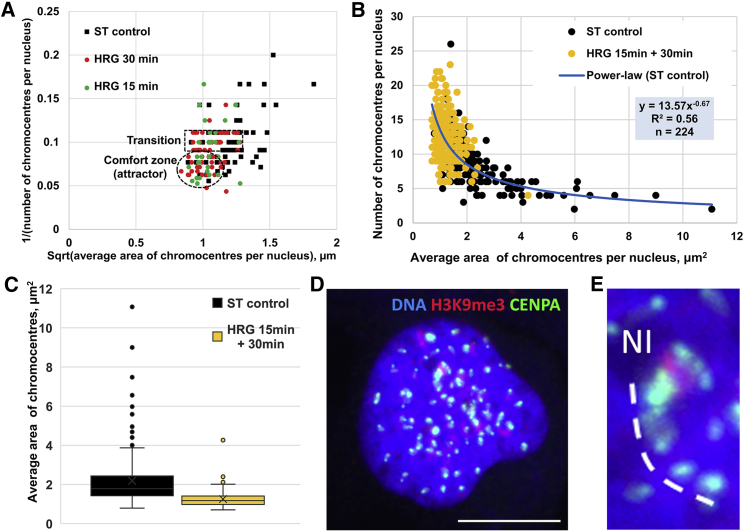
Influence of HRG treatment (15 + 30 min) on PAD distribution in ST control cells. (*A*) Distribution of the average PAD number (inverted) and the perimeter size (square root of the area) measured in one representative experiment and shown separately for samples of 15 and 30 min of HRG treatment. It shows the HRG comfort attractor (*encircled*) achieved already at 15 min of HRG treatment and persisting at 30 min of HRG treatment and the transition zone (*boxed*), which suggests the pairwise fluctuations between sizes of small PADs; (*B*) the relationship between the average number of PADs and their average area per cell; the distribution clearly shows the transition of both HRG 15- and 30-min-cells covering an only small area in ST population. The data of three experiments are merged. ST control cells show a power-law distribution with high correlation (*blue trendline*); (*C*) box plots of the data presented in panel (*B*) showing high heterogeneity of the PADs in ST control cells and low heterogeneity and lower average area in HRG 15- and 30-min samples; (*D*) the example of a cell nucleus after 15 min of HRG treatment. Scale bar, 10 *μ*m. (*E*) Enlarged fragment from (*D*) with characteristic PAD clusters and centromere chain repulsing from the nucleolus (Nl).

For this study, we selected the interval of 15- to 30-min HRG treatment, around the critical gene expression avalanche found by Tsuchiya et al. (15) and the point of the R/G shift that we determined at the 20-min HRG action. We found, in three experiments, that a substantial proportion of cells shifts toward smaller PAD size and larger number condition after 15 min of HRG treatment and remains as such also after 30 min of HRG treatment (Fig. 6, *A*–*C*). In particular, Fig. 6 *A* of the representative experiment shows the decrease of the average PAD area with much smaller variations as compared to the ST control, pointing to the reach of a “comfort zone” (54) that is achieved at HRG 15 min and sustained at HRG 30 min (encircled). This can be interpreted as an attractor equilibrium state characterized by low correlation and variation (Fig. 6, *B* and *C*; (54)).

This state corresponds to a monocentromeric single PAD, with its linear average size around 1 *μ*m, whereas the transition zone (*boxed* in Fig. 6 *A*) with the size between ∼1 and 1.5 PAD diameters corresponds to two PADs, which are likely fluctuating (splitting, fusing, and splitting again), before lodging as a single PAD into a comfort zone. The proportion of cells with large PAD area after HRG action is correspondingly decreased. The summary of the results of three experiments after 15 and 30 min of HRG application (merging the values for 15 and 30 min of HRG treatment) shown in Fig. 6 *B* confirms the accumulation of the treated cells in the population compartment with small, <2 *μ*m^2^, and more numerous PADs, with reduction of the heterogeneity and very low correlation values, as seen on box plots in Fig. 6 *C*. The control and experiment groups in Fig. 6 *C* are significantly different by *t*-test *p*-value = 8 × 10^−21^. It shows that the median large PADs have unraveled into smaller units after 15 min of HRG treatment and remained there at least until 30 min. The distribution of the histogram for ST control again displays a power-law trend, whereas for HRG samples, a very poor correlation is gained with any fit (data not shown). Confocal microscopy of these samples often showed unraveling of PAD clusters into single-centromeric tandem chains directly from the perinucleolar heterochromatin (Fig. 6, *D* and *E*).

To make a long story short, after 15–30 min of HRG action, the most PADs unclustered up to monocentromeric units and lodged in an equilibrium state, likely topologically unraveling and repulsing from the nucleolus-associated large heterochromatin clusters.

We next wanted to verify our 20-min AO DNA structural test results, whether the unraveling of H3K9me3-labeled PADs after 15 and 30 min of HRG treatment was accompanied by the unfolding of the transcribing H3K4me3-marked euchromatin.

### Unfolding of the active H3K4me3-positive chromatin around the unraveled PADs

We applied immunostaining of the two histone H3 modification markers H3K9me3 and H3K4me3 for the visualization of the PADs and active euchromatin around them after 15–30 min HRG treatment, respectively.

**Figure 7:**
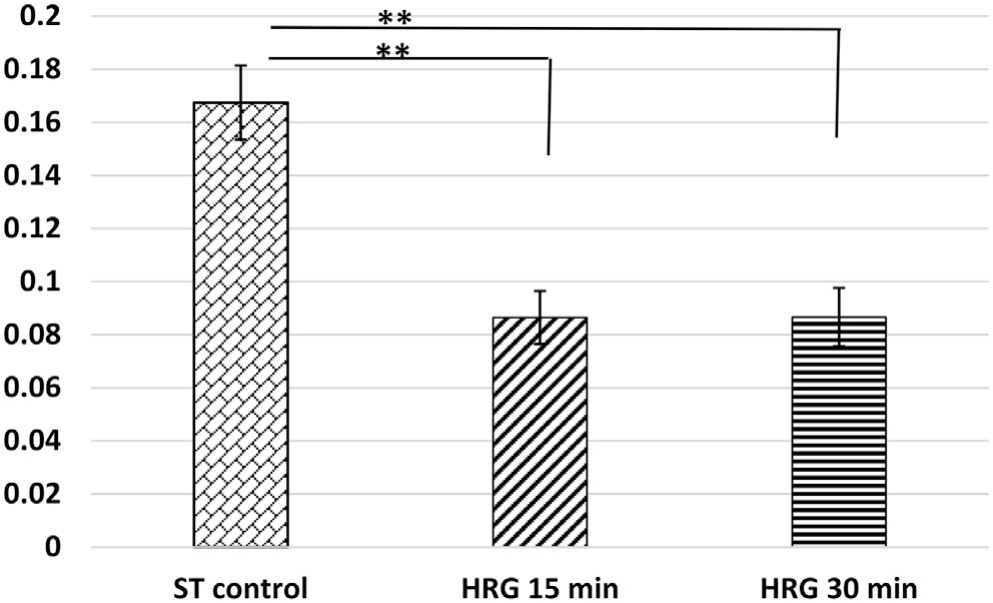
HRG treatment induces unfolding of the H3K4me3-positive chromatin. The results of the measurements of the heterogeneity parameter of the H3K4me3-stained, transcriptionally active chromatin are shown in the green channel using ImagePro Plus 4.5 program. After 15 min of HRG treatment, the active chromatin becomes by half less heterogeneous (smoothened) and remains such at 30 min of HRG treatment. The averages of three independent experiments, with standard deviation.

The distribution of the H3K4me3-stained component was evaluated by imaging with the green optical filter cube (I3) and then measured by the software parameter “heterogeneity” (see in Materials and methods, Microscopy and image analysis) characterizing its smoothness. Indeed, in three independent experiments we found the larger H3K4me3-chromatin heterogeneity in the control cell nuclei with larger chromocenters, whereas in HRG-treated cells, at 15 and 30 min, it was similarly halved (Fig. 7). The data were also confirmed by pilot-selective one-line densitometry through PADs (Fig. S1). It shows denser H3K4me3 “collars” around and admixed to H3K9me3-PADs forming large DAPI-dense chromocenters. On the contrary, in 15-min-HRG-treated samples, this denser H3K4me3-material smoothed out around smaller, multiple clear-cut PADs smearing the contours of DAPI-chromocenters. The H3K4me3 dense “collars” thus represent the facultative heterochromatin unfolding around split PADs which likely lost their position-silencing effect variegation (PEV) effect. Together with AO R/G shift suggesting looser chromatin packaging, this interpretation is in accord with accelerated activation of the several thousand low-transcribed genes found at this time point (15,16).

### qPCR of lncRNA transcripts of two chromosome-specific human centromere satellite 3 DNA: ∼1.5-fold increase after 60 min of HRG treatment at the second step of DNA unfolding

**Figure 8:**
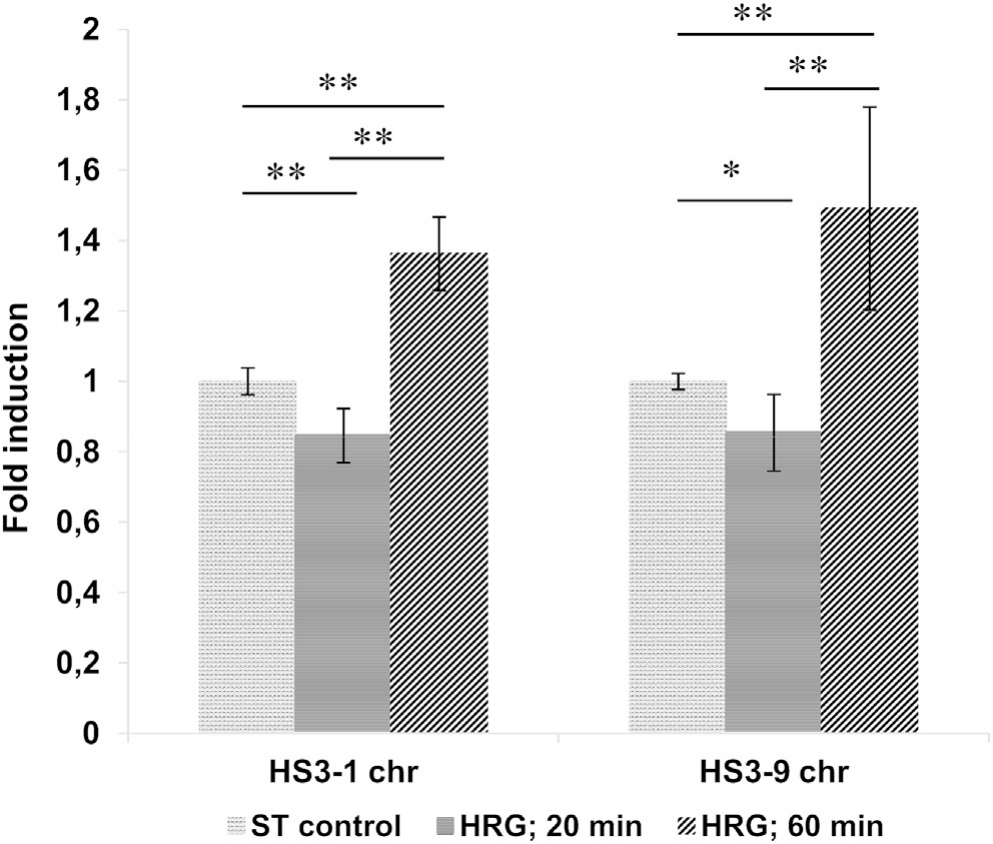
The transcription levels of pericentric HS3 from chr no. 1 and chromosome no. 9, after HRG treatment, were normalized to ST control cells and expressed in averaged fold induction; two independent experiments, four technical replicates; arrow bars represent the standard deviation of all replicates from two experiments, the ST control normalized to one unit. The *p*-values were adjusted by multiple *t*-test correction.

Next, we wished to clarify whether and how the difference in DNA conformation (which by the AO test, revealed two different shifts in DNA unfolding after 20 and 60 min of HRG treatment (Fig. 3)) was associated with possible transcription of lncRNA from the chromosome-specific pericentric minor human satellite 3. The results of the qPCR analysis using chromosome-1- and chromosome-9-specific primers in two independent experiments with four technical replicates in each are presented in Fig. 8.

The results with both chromosome-specific primers show in two experiments the similarly directed changes: the transcription of pericentric HS3 lncRNA at HRG 20 min was decreased by ∼20%, but at HRG 60 min it was ∼1.5-fold increased.

## Discussion

This study aimed to examine whether and how the PADs participate in the very early stage of differentiation called the commitment, which is when cells start changing their fate. In our research of the MCF-7-HRG model, we paid attention to the rearrangements of chromatin and PAD changes at time points when two transcription avalanches have been revealed by two groups in transcriptome studies: between 15 and 20 (15,16) and 60–75 min of HRG action (18), both indicating to SOC as the main driver of the phenomenon at this stage.

The SOC approach, like any statistical mechanics describing the system change occurring nonlinearly “in a jump,” is largely independent of the material composition of the analyzed systems, allowing for a description of phenomena as diverse as earthquakes, solar flares, economic crises, and immune response (53,55). We build upon the hypothesis that the SOC-like features observed for gene expression could have a structural counterpart in PADs dynamics. This aim is achieved by looking for chromatin structural rearrangements at the same time points in which global critical changes in gene expression were observed (15,16,18).

We found for ST MCF-7 cells in the control (most were synchronized to delay in the G0-G1 phase) that they displayed a typical scale-free distribution of PADs consistent with SOC (53). This kind of scaling, most frequent in nature points to a different physical process as compared with Gaussian distribution that presumes the presence of a typical (average) size with a bell-shaped distribution of departures from average.

In the studied cell model, such 1/f correlation allows a critical transition embracing the entire system and corresponds to the fact that the cells convert a constant amount of the pericentric heterochromatin by clustering and unclustering of their PADs from larger units into smaller ones and vice versa. This implies that all these units are interacting as a self-contained system driven by a common physical process (like pulsing) (56), implying the presence of a suprachromosomal network. This puts the genome organization “on the edge of chaos” making it possible to undergo cell fate change by global rewiring through self-organization (20,57,58). The presence of 1/f scaling (or equivalently n^−1^ as we stated in the text) pointing to a decreasing frequency at the increasing size, shared by power-law and/or exponential distributions, supports the continuous aggregation-disaggregation dynamics of PADs allowing for nuclear architecture restructuring.

Although the PADs belong to individual chromosomes with one centromere they act as a system of equivalent units just at the suprachromosomal level of organization. This feature was disclosed because of the methodological solution that we applied to average PADs per cell and thus deprive them of the chromosomal individuality. This methodology resulted in the power-law distribution between PAD size and number. These observables of PADs are strictly linked to the plasticity of this compartment. Reducing the individuality to systemic functionality, the scale-free distribution, in general, was confirmed by the fact that it occurred on different levels of resolution, e.g., in epifluorescent or confocal microscopy measurements, which revealed the finest PADs.

As a consequence, the PADS unraveled at 15 min largely to one-centromere entities. In fact, this means the breakage of positional information of the suprachromosomal network, enabling genome rewiring for phenotype repatterning. The disruption of positional information was mirrored by the drastic decrease in the heterogeneity of H3K4me3-positive chromatin spatial distribution at the transition point (Fig. 7). The generalized decrease of heterogeneity affecting all the genome suggests the entire system can synchronize by bursting in an all-or-none manner (56). That is namely what we have recorded as occurring with PADs, chromatin topology, and gene activity upregulation after 15 min of HRG treatment. Our observations are thus consistent with the notion of SOC with spatial degrees of freedom naturally evolving toward a self-organized critical point (59).

**Figure 9:**
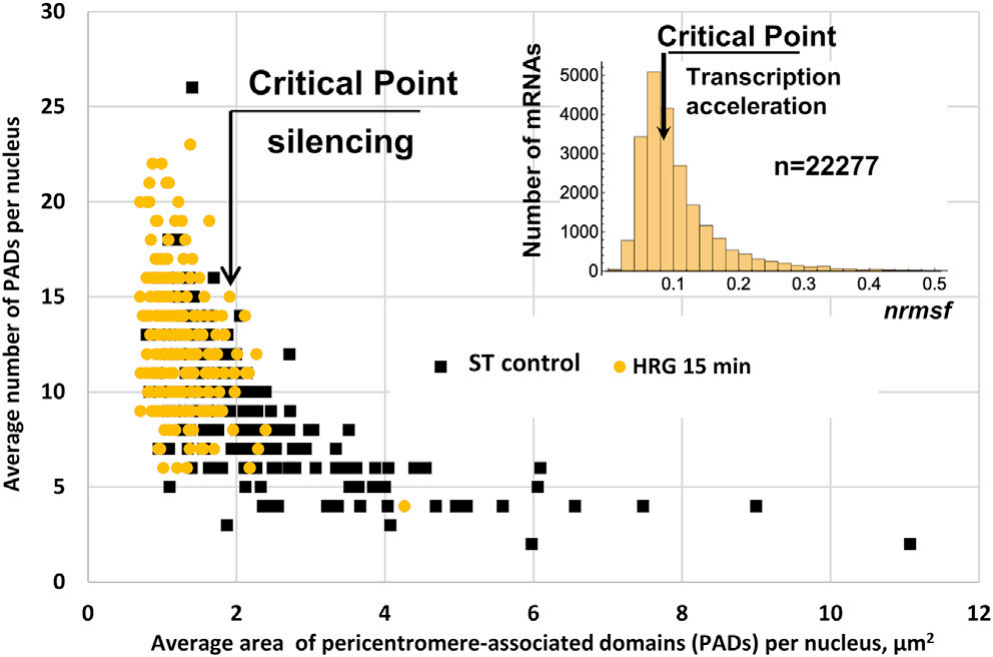
Schematic correlation of data indicating the hypothesis of functional dependency of the chromatin reorganization and gene expression. The commitment of differentiation in MCF-7 cells after 15 min of HRG treatment. Unfolding of PADs under the critical threshold of silencing inducing critical acceleration of the whole-genome transcription is shown. Figure fragment for transcriptome data republished from (16) representing transcription speed change (nrmsf) of 22,277 genes shows the critical point at the 15 min of HRG treatment.

The action of HRG after 15 min caused this self-organizing leap; most PADs unraveled up to the smallest monocentromeric units that were set in the so-called comfort zone at the low-energy equilibrium attractor (with low variation of the uniform declustered PADs). The time window of the PADs’ leap coincided with the window of the critical transition of the first transcription avalanche producing a supercritical “genome engine” reported by Tsuchiya and colleagues (15,16,60), which we also confirmed by a twofold decrease of heterogeneity of the H3K4me3-stained chromatin, which is likely caused by activation and disjoining from PADs of facultative heterochromatin, coinciding with the loss of PAD variance. As well as in support, the first DNA chromatin unfolding indicated by a shift in the AO-structural test (a sign of looser DNA histone interactions) was occurring at the same time interval. So, it may be logical to suggest a coupling of both events PADs splitting under their silencing threshold size (around 1 *μ*m^2^) would allow a first global change of the whole-genome architecture erasing the positional information to occur. An indirect proof in favor of this assumption is the transcription acceleration of sthousands of low-expressed genes (15) likely released from the PEV by PAD bursting. PEV was considered as, functionally, the most important type of positioning effect in the cell nucleus (58). Our hypothesis based on the scale-free critical phase transitions induced as the first step of differentiation commitment is schematically presented in Fig. 9.

Our observations show a directed redistribution from the nucleolus to the nuclear periphery by unraveling (repulsing) PADs. They are well consistent with the capability of the NORs to cluster with large heterochromatic regions of other human chromosomes, which has been known since observations by Susumu Ohno and colleagues (61,62). The finding by the centromere cluster analysis of the tentative NORs boundary of PADs unraveling (radial pulsing) is intriguing in accord with the studies on the relationship between NORs and chromosome territories as a global interactive network for cell nucleus functional organization (63,64) involving intracellular nucleolar phase transitions (65). It is reasonable to note that just the contrary phenomenon (i.e., a pairwise fusion of small post-G2 chromocenters withdrawing the euchromatin DNA and contracting radially toward the central nucleolus) was found in the terminal programmed transcription cessation by the chick-embryonal hypertrophic chondrocytes (19,66).

It is also worth mentioning that, as found earlier, the first genome expression transition at 15–20 min of HRG treatment of MCF-7 cells coinciding with FOS upregulation peak was shown as being performed by the ∼1 Mbp-sized units, corresponding the TADs (15). Our DNA AO study revealed the clear DNA conformational difference between this first transition coinciding with FOS peak and a second one, for more open conformation, after 60 min coinciding with FOSL1 and MYC maximal upregulation. The first transition is mandatory; as we found, it is associated with physical PADs bursting. In turn, the second transition was likely depending on the cooperative energy of individual cells, as seen in three AO experiments. A high cell variability, presence of “rebellious cells,” and involvement of the paracrine feedback mechanisms in the differentiation time course (up to 7 days) were also described in similar models (67,68). Such unstable transitory states are sensitive to external stimuli and cells are searching for a compromise between instructive and permissive paths (69). The second transition in our model involves transcription upregulation of the pericentric lncRNA and the second peak of the early gene stress response. In accordance with our observations, the previous studies on this model revealed that the sustainability of the early response gene network activation starting from 20 min and peaking at 1–1.5 h is needed for initiation of differentiation; it is dependent on the sequential induction by FOS of FOSL1 (FRA-1) downregulated at 3 h by FHL2 (12). We could confirm this biphasic induction in the indicated terms. FOS, the main component of the AP-1 transcription complex (with JUN-family) activated by HRG at 20 min is known to increase the accessibility of the open chromatin (70). Here, we can suggest that this ability may be associated with the observed PADs bursting. In turn, the target of FOS-FOSL1, activated ad maximum at 1 h is likely a universal mediator of the tissue differentiation-specific inducers. In particular to MCF7 cells, it mediates the estrogen receptor-FOSL1 reciprocal signaling (ER is binding to FOSL1 promoter) (71). FOS/FOSL1 complex was reported to be indispensable in embryonic development and trophoblast, osteoclast, epidermis, bone, and fat differentiation (72, 73, 74). These transcription factors binding DNA by zinc finger-helix domains contain in their promoters the epigenetically bivalent chromatin (75), which capable of exerting rapid activation of a poised gene for cell fate change. In turn, their C-terminus contain intrinsically disordered domains (76,77), enabling the promiscuous links with various molecular targets. In addition, the known FOS-dependent activation of MYC (12,45) found by us with a maximum at 1 h, can also contribute to the accessibility of the open chromatin by acetylation of lysine in histones H3 and H4 tails including facultative heterochromatin (78) and opening the window of the genome multipotency (79).

All these factors starting at 15 min of HRG treatment with the physical bursting of pericentric heterochromatin, which likely lifts the genome-wide bar of genes silencing, destroys the suprachromosomal network and erases epigenetic formula by all-or-none dynamics. The early stress response genes particularly designed for cell fate change are involved. Both components cooperatively achieve the commitment of genome rewiring for differentiation repatterning. In line with our data (Fig. 2), this new stable state seen from the cessation of biphasic early gene response and transcription turbulency is set in this model by 3 h after HRG treatment (12,16).

## Conclusion

The novelty of this study relies on the definition of the role of PADs in the differentiation initiation (commitment) for cell fate change occurring within 1 h after treatment of MCF7 breast cancer with HRG. The differentiation commitment is likely achieved by the unraveling of PADs under the threshold of silencing at HRG 15–20 min, starting and transferring the momentum energy to TADs and ensuring their unfolding for further transcription activation at 60 min. The first and second steps are likely tightly coupled by the intensity and sustainability of the early gene response in the whole cell population. On a more general ground, as aptly stressed in (55) rapid phase transition and phase separation as well as their tipping points are penetrating phenomena in biology and are intrinsic properties of biological systems ranging from basic molecule complexes to cells and all way up to entire ecosystems. Along this line of thinking, we could conclude that the reversible unclustering of PADs represents the mesoscale critical phase separation that acts as the material basis of the critical transition observed at gene expression. It is worth stressing the “hypothesis-generation” more than “hypothesis-testing” character of our study that mainly stems from the temporal synchronization of events happening at nuclear organization and gene expression levels. Nevertheless, these preliminary findings open the way to new therapeutic strategies impinging on the gene networks behavior and ask for future studies investigating the link between whole-genome transitions and cell fate change going beyond the reductionist paradigm.

## Author contributions

Conceptualization, J.K., T.F., J.E., M.H., and A.G.; methodology, J.E., T.F., J.K., M.H., and A.G.; investigation, J.K., K.S., T.F., I.I., F.R., P.Z., J.E., and M.H.; validation, P.Z., T.F., J.K., K.S., and F.R.; formal analysis, P.Z. and J.K.; writing (original draft preparation), J.E., K.S., and M.H.; writing (review and editing), J.E., J.K., I.I., M.H., K.S., T.F., and A.G.; visualization, K.S., J.K., J.E., and P.Z.; supervision, J.E.; funding acquisition, K.S., J.E., M.H., and J.E. All authors have read and agreed to the published version of the manuscript.

## Supporting material

Document S1. Figure S1

Document S2. Article plus supporting material

## References

[1] Erenpreisa J., Giuliani A. (2019). Resolution of complex issues in genome regulation and cancer requires non-linear and network-based thermodynamics. Int. J. Mol. Sci.

[2] Lacroix M., Leclercq G. (2004). Relevance of breast cancer cell lines as models for breast tumours: an update. Breast Cancer Res. Treat.

[3] Comşa Ş., Cîmpean A.M., Raica M. (2015). The story of MCF-7 breast cancer cell line: 40 years of experience in research. Anticancer Res.

[4] Dai X., Cheng H., Li J. (2017). Breast cancer cell line classification and its relevance with breast tumor subtyping. J. Cancer.

[5] Montero J.C., Seoane S., Pandiella A. (2011). P-Rex1 participates in Neuregulin-ErbB signal transduction and its expression correlates with patient outcome in breast cancer. Oncogene.

[6] Nagashima T., Shimodaira H., Hatakeyama M. (2007). Quantitative transcriptional control of ErbB receptor signaling undergoes graded to biphasic response for cell differentiation. J. Biol. Chem.

[7] Britsch S. (2007). The neuregulin-I/ErbB signaling system in development and disease. Adv. Anat. Embryol. Cell Biol.

[8] Zhu W.-Z., Xie Y., Laflamme M.A. (2010). Neuregulin/ErbB signaling regulates cardiac subtype specification in differentiating human embryonic stem cells. Circ. Res.

[9] Wang Y.-N., Lee H.-H., Hung M.-C. (2012). Membrane-bound trafficking regulates nuclear transport of integral epidermal growth factor receptor (EGFR) and ErbB-2. J. Biol. Chem.

[10] Pilarczyk G., Nesnidal I., Hausmann M. (2017). Localisation microscopy of breast epithelial ErbB-2 receptors and gap junctions: trafficking after γ-irradiation, neuregulin-1β, and trastuzumab application. Int. J. Mol. Sci.

[11] Hao J., Galindo C.L., Sawyer D.B. (2014). Neuregulin-1β induces embryonic stem cell cardiomyogenesis via ErbB3/ErbB2 receptors. Biochem. J.

[12] Saeki Y., Endo T., Okada-Hatakeyama M. (2009). Ligand-specific sequential regulation of transcription factors for differentiation of MCF-7 cells. BMC Genomics.

[13] Wang Y., Prywes R. (2000). Activation of the c-fos enhancer by the erk MAP kinase pathway through two sequence elements: the c-fos AP-1 and p62TCF sites. Oncogene.

[14] Nakakuki T., Birtwistle M.R., Kholodenko B.N. (2010). Ligand-specific c-Fos expression emerges from the spatiotemporal control of ErbB network dynamics. Cell.

[15] Tsuchiya M., Giuliani A., Yoshikawa K. (2015). Emergent self-organized criticality in gene expression dynamics: temporal development of global phase transition revealed in a cancer cell line. PLoS One.

[16] Tsuchiya M., Giuliani A., Yoshikawa K. (2016). Self-organizing global gene expression regulated through criticality: mechanism of the cell-fate change. PLoS One.

[17] Zimatore G., Tsuchiya M., Giuliani A. (2019). Self-organization of whole gene expression through coordinated chromatin structural transition: validation of self-organized critical control of genome expression. bioRxiv.

[18] Chen P., Liu R., Aihara K. (2015). Identifying critical differentiation state of MCF-7 cells for breast cancer by dynamical network biomarkers. Front. Genet.

[19] Erenpreisa J., Zhukotsky A. (1993). Interphase genome as the active space: chromatin dynamics during chick embryo chondrogenesis. Mech. Ageing Dev.

[20] Misteli T. (2001). The concept of self-organization in cellular architecture. J. Cell Biol.

[21] Yoshikawa K. (2002). Field hypothesis on the self-regulation of gene expression. J. Biol. Phys.

[22] Erenpreisa J., Zhukotsky A. (1992). The genome field. *Proc*. *Latv*. *Acad*. *Sci. Ser. B*.

[23] Boutanaev A.M., Mikhaylova L.M., Nurminsky D.I. (2005). The pattern of chromosome folding in interphase is outlined by the linear gene density profile. Mol. Cell. Biol.

[24] Cavalli G. (2007). Chromosome kissing. Curr. Opin. Genet. Dev.

[25] Lieberman-Aiden E., van Berkum N.L., Dekker J. (2009). Comprehensive mapping of long-range interactions reveals folding principles of the human genome. Science.

[26] Cabianca D.S., Muñoz-Jiménez C., Gasser S.M. (2019). Active chromatin marks drive spatial sequestration of heterochromatin in *C. elegans* nuclei. Nature.

[27] Erdel F., Rippe K. (2018). Formation of chromatin subcompartments by phase separation. Biophys. J.

[28] Solovei I., Thanisch K., Feodorova Y. (2016). How to rule the nucleus: divide et impera. Curr. Opin. Cell Biol.

[29] Hancock R., Jeon K.W. (2014). Preface. New models of the cell nucleus: crowding, entropic forces, phase separation, and fractals. Int. Rev. Cell Mol. Biol.

[30] Beil M., Dürschmied D., Irinopoulou T. (2002). Spatial distribution patterns of interphase centromeres during retinoic acid-induced differentiation of promyelocytic leukemia cells. Cytometry.

[31] Ostromyshenskii D.I., Chernyaeva E.N., Podgornaya O.I. (2018). Mouse chromocenters DNA content: sequencing and in silico analysis. BMC Genomics.

[32] Bártová E., Kozubek S., Hausmann M. (2002). Nuclear structure and gene activity in human differentiated cells. J. Struct. Biol.

[33] Wijchers P.J., Geeven G., de Laat W. (2015). Characterization and dynamics of pericentromere-associated domains in mice. Genome Res.

[34] Probst A.V., Almouzni G. (2008). Pericentric heterochromatin: dynamic organization during early development in mammals. Differentiation.

[35] Solovei I., Schermelleh L., Cremer T. (2004). Differences in centromere positioning of cycling and postmitotic human cell types. Chromosoma.

[36] Kobayakawa S., Miike K., Abe K. (2007). Dynamic changes in the epigenomic state and nuclear organization of differentiating mouse embryonic stem cells. Genes Cells.

[37] Hartley G., O’Neill R.J. (2019). Centromere repeats: hidden gems of the genome. Genes (Basel).

[38] Meshorer E., Yellajoshula D., Misteli T. (2006). Hyperdynamic plasticity of chromatin proteins in pluripotent embryonic stem cells. Dev. Cell.

[39] Hausmann M., Ilić N., Blank M. (2017). Challenges for super-resolution localization microscopy and biomolecular fluorescent nano-probing in cancer research. Int. J. Mol. Sci.

[40] Vandesompele J., De Preter K., Speleman F. (2002). Accurate normalization of real-time quantitative RT-PCR data by geometric averaging of multiple internal control genes. Genome Biol.

[41] Morozov V.M., Gavrilova E.V., Ishov A.M. (2012). Dualistic function of Daxx at centromeric and pericentromeric heterochromatin in normal and stress conditions. Nucleus.

[42] Enukashvily N.I., Donev R., Podgornaya O.I. (2007). Human chromosome 1 satellite 3 DNA is decondensed, demethylated and transcribed in senescent cells and in A431 epithelial carcinoma cells. Cytogenet. Genome Res.

[43] Benjamini Y., Hochberg Y. (1995). Controlling the false discovery rate: a practical and powerful approach to multiple testing. J. R. Stat. Soc. B.

[44] Sinclair J.D. (1988). Multiple t-tests are appropriate in science. Trends Pharmacol. Sci.

[45] Vartanian R., Masri J., Gera J. (2011). AP-1 regulates cyclin D1 and c-MYC transcription in an AKT-dependent manner in response to mTOR inhibition: role of AIP4/Itch-mediated JUNB degradation. Mol. Cancer Res.

[46] Darzynkiewicz Z. (1990). Differential staining of DNA and RNA in intact cells and isolated cell nuclei with acridine orange. Methods Cell Biol.

[47] Dobrucki J., Darzynkiewicz Z. (2001). Chromatin condensation and sensitivity of DNA in situ to denaturation during cell cycle and apoptosis--a confocal microscopy study. Micron.

[48] Erenpreĭsa E.A., Sondore O. Iu. (1989). The relation of the phenomenon of nuclear achromasia to cell activation for proliferation. Eksp. Onkol.

[49] Erenpreisa J., Freivalds T., Alston R. (1997). Apoptotic cell nuclei favour aggregation and fluorescence quenching of DNA dyes. Histochem. Cell Biol.

[50] Erenpreisa J., Krigerts J., Freivalds T. (2018). Differential staining of peripheral nuclear chromatin with acridine orange implies an A-form epichromatin conformation of the DNA. Nucleus.

[51] Evenson D., Darzynkiewicz Z., Ballachey B. (1986). Changes in accessibility of DNA to various fluorochromes during spermatogenesis. Cytometry.

[52] Erenpreiss J., Bars J., Zalkalns J. (2001). Comparative study of cytochemical tests for sperm chromatin integrity. J. Androl.

[53] Marković D., Gros C. (2014). Power laws and self-organized criticality in theory and nature. Phys. Rep.

[54] Gorban A.N., Smirnova E.V., Tyukina T.A. (2010). Correlations, risk and crisis: from physiology to finance. Physica A Stat. Mech. Appl.

[55] Wang P., Chen L. (2020). Critical transitions and tipping points in EMT. Quant. Biol.

[56] Batista C.A.S., Batista A.M., Viana R.L. (2009). Bursting synchronization in scale-free networks. Chaos Solitons Fractals.

[57] Kauffman S. (1996). At Home in the Universe: The Search for the Laws of Self-Organization and Complexity.

[58] Misteli T. (2005). Concepts in nuclear architecture. BioEssays.

[59] Bak P., Tang C., Wiesenfeld K. (1987). Self-organized criticality: an explanation of the 1/f noise. Phys. Rev. Lett.

[60] Tsuchiya M., Giuliani A., Yoshikawa K. (2020). Cell-fate determination from embryo to cancer development: genomic mechanism elucidated. Int. J. Mol. Sci.

[61] Ohno S., Trujillo J.M., Kinosita R. (1961). Nucleolus-organisers in the causation of chromosomal anomalies in man. Lancet.

[62] Stahl A., Hartung M., Fouet C. (1976). Chromosomal constitution of nucleolus-associated chromatin in man. Hum. Genet.

[63] Iarovaia O.V., Minina E.P., Vassetzky Y.S. (2019). Nucleolus: a central hub for nuclear functions. Trends Cell Biol.

[64] Pliss A., Fritz A.J., Berezney R. (2015). Non-random patterns in the distribution of NOR-bearing chromosome territories in human fibroblasts: a network model of interactions. J. Cell. Physiol.

[65] Weber S.C., Brangwynne C.P. (2015). Inverse size scaling of the nucleolus by a concentration-dependent phase transition. Curr. Biol.

[66] Erenpreisa J., Zhukotsky A., Kozlov A. (1993). The chromatin network: image analysis of differentiating chick embryo chondrocytes. Eur. J. Histochem.

[67] Mojtahedi M., Skupin A., Huang S. (2016). Cell fate decision as high-dimensional critical state transition. PLoS Biol.

[68] Stumpf P.S., Smith R.C.G., MacArthur B.D. (2017). Stem cell differentiation as a non-markov stochastic process. Cell Syst.

[69] Bizzarri M., Giuliani A., Cucina A. (2020). Constraints shape cell function and morphology by canalizing the developmental path along the Waddington’s landscape. BioEssays.

[70] Biddie S.C., John S., Hager G.L. (2011). Transcription factor AP1 potentiates chromatin accessibility and glucocorticoid receptor binding. Mol. Cell.

[71] Chen C., Li C., Zhao A. (2020). Estrogen-induced FOS-like 1 regulates matrix metalloproteinase expression and the motility of human endometrial and decidual stromal cells. J. Biol. Chem.

[72] Matsuo K., Owens J.M., Wagner E.F. (2000). Fosl1 is a transcriptional target of c-Fos during osteoclast differentiation. Nat. Genet.

[73] Kubota K., Rumi M.A.K., Soares M.J. (2012). FOSL1 regulation of trophoblast cell differentiation: partners and gene targets. Biol. Reprod.

[74] Eckert R.L., Adhikary G., Rorke E.A. (2013). AP1 transcription factors in epidermal differentiation and skin cancer. J. Skin Cancer.

[75] Court F., Arnaud P. (2017). An annotated list of bivalent chromatin regions in human ES cells: a new tool for cancer epigenetic research. Oncotarget.

[76] Campbell K.M., Terrell A.R., Lumb K.J. (2000). Intrinsic structural disorder of the C-terminal activation domain from the bZIP transcription factor Fos. Biochemistry.

[77] van der Lee R., Buljan M., Babu M.M. (2014). Classification of intrinsically disordered regions and proteins. Chem. Rev.

[78] Martinato F., Cesaroni M., Guccione E. (2008). Analysis of Myc-induced histone modifications on target chromatin. PLoS One.

[79] Yoshida G.J. (2018). Emerging roles of Myc in stem cell biology and novel tumor therapies. J. Exp. Clin. Cancer Res.

