# Supplementary material for: Differentiating cancer cells reveal early large-scale genome regulation by pericentric domains
Source: Biophys J. 2021 Jan 14;120(4):711–24. doi: 10.1016/j.bpj.2021.01.002 (PMC7896032; doi:10.1016/j.bpj.2021.01.002)
Supplement: Document S1. Figure S1 [file mmc1.pdf]

Biophysical Journal, Volume *120*

Supplemental Information

Differentiating cancer cells reveal early large-scale genome regulation by pericentric domains

Jekabs Krigerts, Kristine Salmina, Talivaldis Freivalds, Pawel Zayakin, Felikss Rumnieks, Inna Inashkina, Alessandro Giuliani, Michael Hausmann, and Jekaterina Erenpreisa

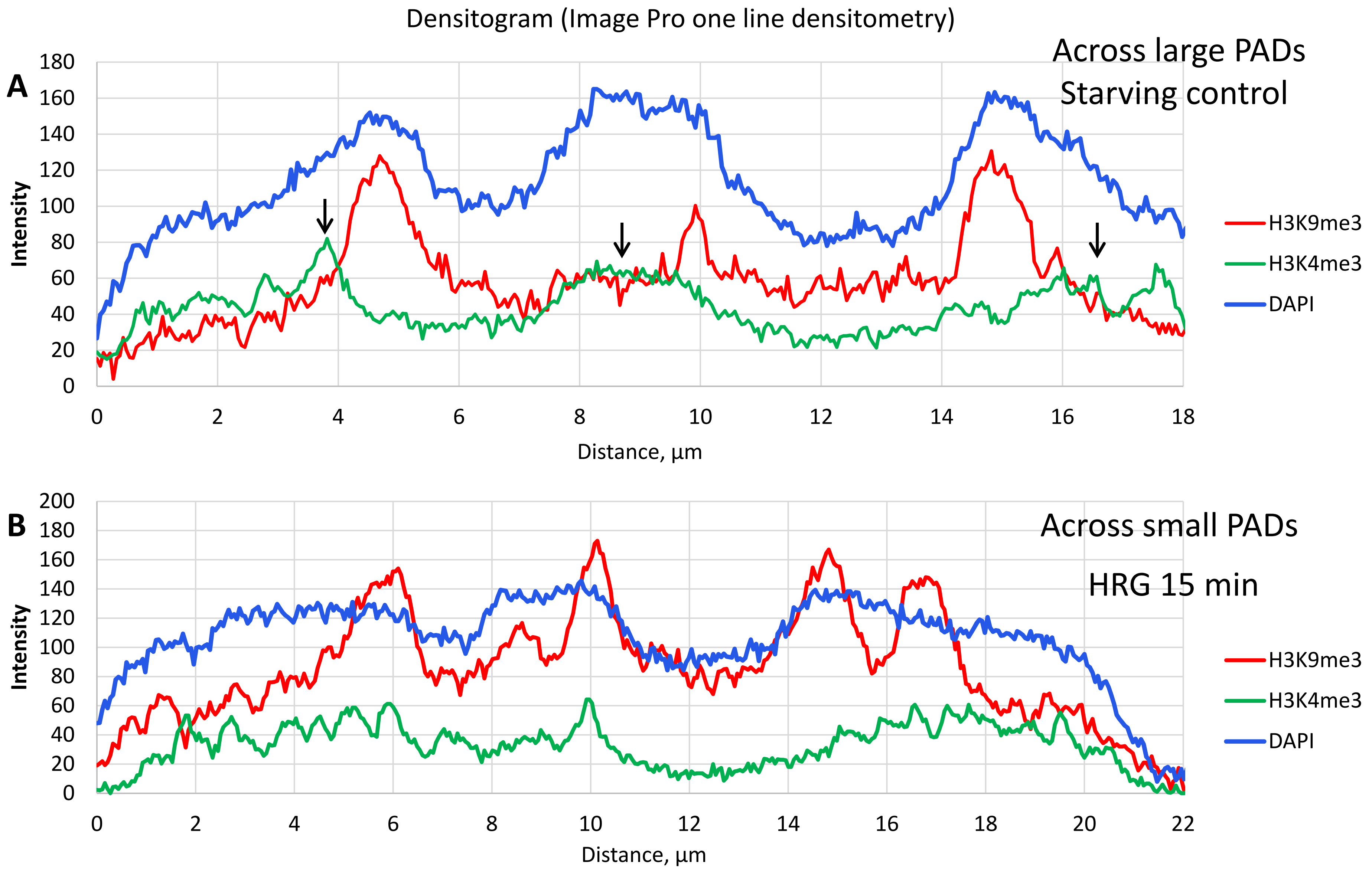

Figure S1. Three-line mean densitometry examples across : (A) large PADs in starving control and (B) small PADs at HRG-15 min in the double-immunostained **H3K9me3**/**H3K4me3** chromatin, counterstained with **DAPI** (imaged in corresponding optical channels) reveal in (A) the relatively dense **H3K4me3** "collars" (arrows) at the borders and admixed to **PADs** included in larger **DAPI**-dense chromocentres, while in (B) the **H3K4me3**-positive material seems repulsed from **PADS** and distributed more evenly, decreasing the density of the **DAPI-** chromocentres.
